# Comparison of Smoothing Filters in Analysis of EEG Data for the Medical Diagnostics Purposes

**DOI:** 10.3390/s20030807

**Published:** 2020-02-02

**Authors:** Aleksandra Kawala-Sterniuk, Michal Podpora, Mariusz Pelc, Monika Blaszczyszyn, Edward Jacek Gorzelanczyk, Radek Martinek, Stepan Ozana

**Affiliations:** 1 Faculty of Electrical Engineering, Opole University of Technology, Automatic Control and Informatics, 45-758 Opole, Poland; m.podpora@po.edu.pl (M.P.); m.pelc@greenwich.ac.uk (M.P.); 2 Department of Computing and Information Systems, University of Greenwich, SE10 9LS London, UK; 3 Faculty of Physical Education and Physiotherapy, Opole University of Technology, 45-758 Opole, Poland; m.blaszczyszyn@po.edu.pl; 4 Department of Theoretical Basis of BioMedical Sciences and Medical Informatics, Nicolaus Copernicus University, Collegium Medicum, 85-067 Bydgoszcz, Poland; medsystem@medsystem.com.pl; 5 Institute of Philosophy, Kazimierz Wielki University, 85-092 Bydgoszcz, Poland; 6 Babinski Specialist Psychiatric Healthcare Center, Outpatient Addiction Treatment, 91-229 Lodz, Poland; 7 The Society for the Substitution Treatment of Addiction “Medically Assisted Recovery”, 85-791 Bydgoszcz, Poland; 8 Department of Cybernetics and Biomedical Engineering, VSB-Technical University Ostrava, FEECS, Ostrava-Poruba 708 00, Czech Republic; radek.martinek@vsb.cz

**Keywords:** electroencephalography, signal processing, smoothing filters, Savitzky–Golay filter

## Abstract

This paper covers a brief review of both the advantages and disadvantages of the implementation of various smoothing filters in the analysis of electroencephalography (EEG) data for the purpose of potential medical diagnostics. The EEG data are very prone to the occurrence of various internal and external artifacts and signal distortions. In this paper, three types of smoothing filters were compared: smooth filter, median filter and Savitzky–Golay filter. The authors of this paper compared those filters and proved their usefulness, as they made the analyzed data more legible for diagnostic purposes. The obtained results were promising, however, the studies on finding perfect filtering methods are still in progress.

## 1. Introduction

The human brain is one of the most important organs, which is responsible for electrical signals’ transmission all over the body and is mainly controlled by the nervous system. The brain-generated electrical data is known as electroencephalograph (EEG) [1,2].

Analysis of biomedical data has been the subject of analysis for numerous researchers all over the world, despite its challenging nature. This is because they are frequently riddled with various internal and external artifacts such as high-frequency noise, or in case of EEG, by another biomedical signal such as electromyography (EMG), electrocardiography (ECG) or electrooculography (EOG) [3,4,5,6]. Not only do other (stronger) biomedical signals affect the EEG signals, but so do numerous external artifacts [6,7,8].

Electroencephalography also provides manifold useful information regarding inter alia various psychological dysfunctions or mental illnesses and even hints on how the mind works [9,10].

This paper is a review of the implementation of various smoothing filters in order to show the efficiency and usability of such in the filtering of biomedical data. The authors of this work focus on the evaluation of both advantages and disadvantages of the above-mentioned methods and their potential implementation in the analysis of EEG signals [6,11,12].

The interdisciplinary character of this work proves the necessity of cooperation of various scientific areas in data analysis. Mathematical methods enable to inter alia predict growth of tumor [12] or design appropriate filters in order to remove artifacts affecting bio-medical data [13,14].

The human brain starts its neural activity during the second pregnancy trimester, between the 17th and 23rd week. The electrical activity of the human brain represents not only state of the brain but also the condition of the whole body [9,10].

The history of electroencephalography started in the 19th century, where Carlo Matteucci and Emil Du Bois-Reymond were the first people who registered the electrical signals from muscle nerves using a very basic galvanometer. The first person who decided to place two electrodes connected to the galvanometer on the scalp was Richard Caton (in 1875) [10,15]. The father of electroencephalography is Hans Berger, who, nearly 50 years later, recorded first proper human signals [15,16].

Hans Berger started his research on human EEG already in 1920 and published his first report in 1929, he was able to distinguish the alpha frequencies as the major EEG component [10,16]. He was the first one to record sleep spindles and to observe the hypoxia effect on the human brain or nature of some brain disorders such as epilepsy. He was also interested in brain tumors and was the first one to find a correlation between mental activities and the changes in the EEG signals [10]. The brain can work due to information coming from the body’s organs (such as the circulatory system, digestive system, genitourinary system, skin).

The authors of this paper focus mainly on electroencephalography as a non-invasive method of brain activity measurement and the obtained data is very useful not only to diagnose but also to monitor various disorders such as inter alia head trauma, tumors, epilepsy, sleep problems [17,18,19].

The EEG-based analysis is also inexpensive, safe and easy to carry out [18,20]. Most of the modern devices are also portable which makes it a very popular solution for brain–computer interfaces [21,22]. Modern EEG can be a good alternative to magnetic resonance imaging (MRI) or computer tomography (CT) [19,20].

All the above has led the electroencephalography to be the most frequently used method for neuro-imaging especially in the past twenty years [6,19,23].

The main aim of this paper was to test and compare various filters with smoothing features, such as median filter and Savitzky–Golay filter and of course classic ’smooth’ filter. Smoothing EEG data enables to observe i.e., some action and the process of smoothing usually does not affect the signal in a way traditional filtering does.

## 2. Methods

Based on literature review and authors’ experience, it is important to choose appropriate filtering methods, so that important information would not be lost. Some work, where a similar study was performed, suggest choosing (while filtering using Savitzky–Golay filters) as high as possible window length *N* and as low as possible frame *L* [24]. However, the tests carried out by the authors of this paper proved that higher *N* value smoothed the signals too strongly and the obtained results were less legible and less useful for the diagnostics’ purposes.

For the purposes of this study, the authors applied various smoothing filters. The first one was the basic, classic smoothing filter with the default span for the moving average. The second one was similar, but with the defined moving average parameter of 15. The third one was Savitzky–Golay filter. A generalized moving average with filter coefficients determined by an unweighted linear least-squares regression and a polynomial model of specified degree (the authors decided to apply the default one, which is 2) and it could be accepted as nonuniform predictor data. The 4th smoothing filter was the Savitzky–Golay filter with the following parameters: 4th (order) and 27 (frame length). The last, 5th one was the Median Filter of the 9th order.

The results were satisfying. As mentioned above, Savitzky–Golay is a digital polynomial filter (or a least smoothing filter) [25]. Both filters are smoothing filters [26,27,28]. The classic, ‘basic’ smoothing filter smooths the data in the column vector using a moving average filter, which works in the way that it replaces each data point with the average of the neighbor data points (defined within its span). It is similar to the lowpass filtering.

The authors of this paper decided to focus on four channels only: ‘C3’, ‘C4’, ‘P3’ and ‘P4’, because the ‘C3’ and ‘C4’ electrodes are placed above the primary motor cortex area for the hand and foot movements, which was assumed to be the appropriate location for analysis of hand movements [29,30]. The location of the ‘C3’ and ‘C4’ electrodes is also linked with the motor preparation and movement execution, where the ‘P3’ and ‘P4’ electrodes represent the parietal area and are functionally related to integration of sensory information from different modalities [30,31]. These are also so-called homologous electrode pairs (left-right centrals and left-right parietals) [32].

**Figure 1:**
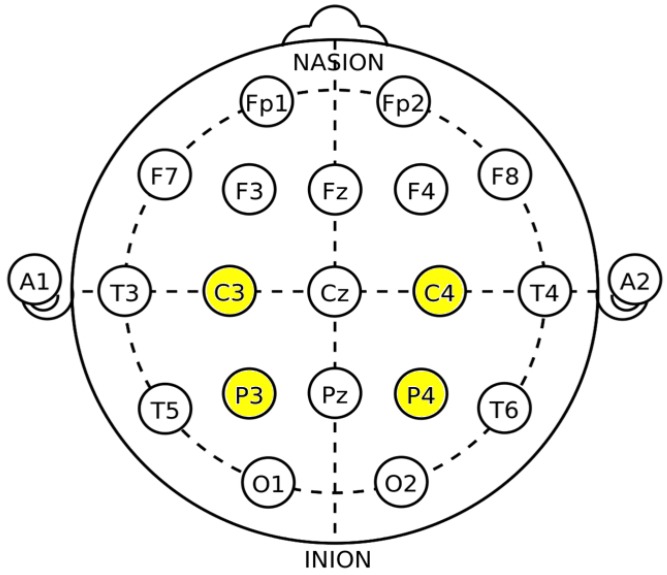
Location of the electrodes placed on scalp.

In Figure 1 location of the analyzed electrodes (based on the 10-20 system) was illustrated.

As it was mentioned above, the ‘C3’ and ‘C4’ locations are located over the contra-lateral cortical regions and these are responsible for the limbs’ movements, especially for the hand movements [33]. These are also linked with motor preparation and execution, while the ‘P3’ and ‘P4’ represent the medial parietal areas linked with the sensory information [30].

Based on thorough literature study and authors’ experience, chasing the ‘C3’, ‘C4’, ‘P3’ and ‘P4’ was caused by their location above particular cortex areas. The most commonly selected areas are of course the ‘C3’ and ‘C4’ for both imagery and real movements, the electrodes from the parietal regions (such as ‘P3’ and ‘P4’) are less frequently used, but their location is also very useful for both imagery and real movements’ classification [34,35].

### Applied Smoothing Filtering

For the purpose of signals’ analysis improvement, the authors decided to perform some tests in the analysis of bio-medical data, in particular, EEG signals, with the implementation of smoothing filters such as Savitzky–Golay, smooth and median filters. This is because smoothing of bio-medical signals require additional attention as the data, in particular, EEG signals, are very sensitive and prone to various artifacts. Some frequency ranges may also contain crucial information, potentially important for diagnostic purposes, and an incorrect choice of processing or filtering methods may affect these [27].

Therefore, smoothing filters have become recently very popular as they enable the extraction of desired data from analyzed signals. The smoothing process modifies the signal’s data points so noises are reduced, and the points that are lower than their neighboring points are increased. It results in a smoother signal [36,37,38,39].

Smoothing of EEG signals plays a crucial role in inter alia diagnostics as it makes the data more legible, therefore the authors of this paper tested various smoothing filters, where each had advantages and disadvantages. Most filters apply averaging in a certain window, such as inter alia Savitzky–Golay or use frequency-domain representation, e.g., Fourier-based filters [28].

This work discusses the advantages and disadvantages of classic smooth filters such as medfilter or Savitzky–Golay filter, which were chosen because they differ from other filters as they reduce the risk of data cutouts, and the smoothed values can be written as a linear transformation of the values. The performed smoothing operation is known as a linear smoother [25,27].

The choice of appropriate filtering is challenging as non-linear filters differ from linear filters, in a way that they could be adaptive. In practice, this means that they retain the so-called edges, which are frequently present in the EEG signals [36,37,38,40,41]. Smoothing filters’ (Savitzky–Golay and Median filters) application enables to correct inter alia spikes present in the data [25,27].

The Savitzky–Golay filter is a least square smoothing filter (digital polynomial filter), its working principles involve replacing each value with a new value, previously obtained from a polynomial fitting, which is performed with a basic linear least-square fitting to the 2n+1 neighboring points, where the value *n* could be equal or greater than the order of the applied polynomial. The more neighbors are applied, the smoother will be the final signal [25]. It smooths the fluctuations and increases the signal–noise ratio (SNR) without significant distortion of the analyzed data [27,42].

The median filter is a non-linear filter, in which the mean value of a sequence (of values in the ascending order of data) of the processed point and its surroundings is measured. The advantage of this filter is that all of the values that deviate from the average are omitted [42]. Such filters are also using–in case of 1D filtering—a window (a sequence of values of the input signal) of a predefined length [43]. The output signal is composed of the individual median values of all the windows. The filter can be applied to offline data using the moving median algorithm, which is similar to the moving average, but for some applications is considered to be better [44] (because it is not averaging the neighboring values), while for some other applications (including trading systems [45]) the moving average is preferred. The median filter can also be easily applied to online data (acquired and processed in realtime) by implementing it in (or as) an intermediate buffer for the measured values.

While the median filter is significantly more efficient in eliminating spikes than moving average and the neighboring values (i.e., these output values-to-be-calculated that happen to have the spike within their input window) are not shifted by the spike. The reason for this is the median filter’s robustness to outliers, whereas the moving average calculates the output value using all window’s values, including the outlier [44].

The simplicity of the median filter’s implementation, as well as its efficiency in filtering spikes and delicate smoothing of the signal, has convinced the authors to choose it as one of the considered filtering options. In most cases, window length of three is enough for basic and simple filtering (if the signal contains one-value spikes), and longer windows are valued for their better smoothing properties, but in the processed EEG signal the spikes were not just single-value outliers, therefore a longer window was needed and used.

## 3. Results

The data applied for this study purpose was downloaded from the open-source database: “GigaScience database, GigaDB” [46]. For the study purposes, the authors of the database tested 52 healthy participants: 19 females (mean age ± SD age = 24.8 ± 3.86 years). They were able to collect 20 trials of real hand movements (left and right hands), two subjects were both-handed. As a result, both EEG and EMG signals were obtained. The data was recorded with the implementation of 64 Ag/AgCl active electrodes placed in accordance with the 10-10 system. The sampling frequency was 512 Hz. The two EMG electrodes were attached to the flexor digitorum profundus and extensor digitorum [46]. The used EEG device was the Biosemi ActiveTwo system, with the BCI2000 system 3.0.2 [47].

**Figure 2:**
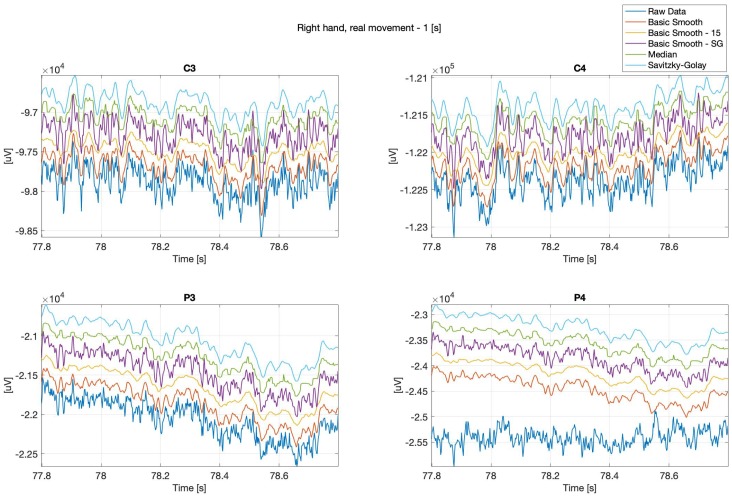
Right hand, real movement, C3, C4, P3 and P4 channels.

**Figure 3:**
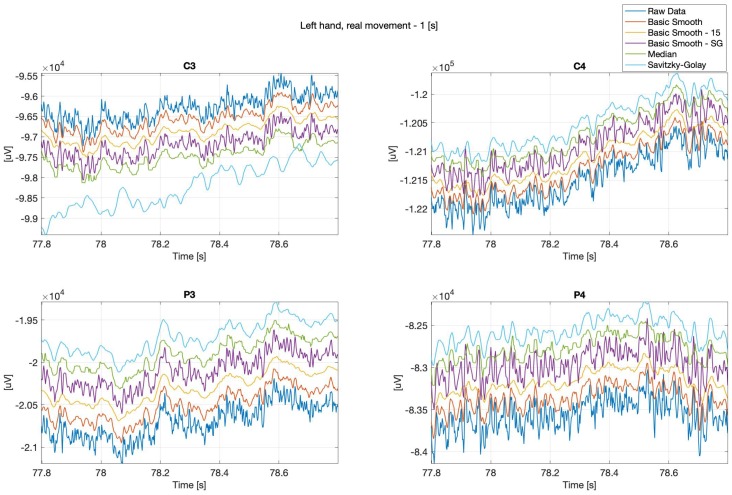
Left hand, real movement, C3, C4, P3 and P4 channels.

Below (see: Figure 2 and Figure 3) are presented sample real hand movements, 1 second intervals only, where Figure 2, illustrates right hand movement and Figure 3 left hand one. The signals were recorded during real limb movements from locations ‘C3’, ‘C4’, ‘P3’ and ‘P4’, while the next series (Figures 8–13) concerns imaginary right and left upper limbs movements recorded in the same location.

It can be observed, that the data “smoothing” affected the overall shape of the signals, the closest to the original one is the data filtered with the basic-smooth-Savitzky–Golay filter.

**Figure 4:**
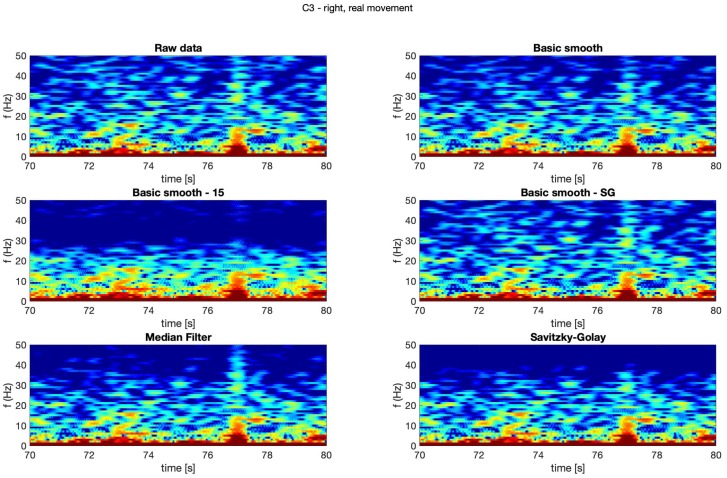
Spectrogram—C3, right hand, real movement.

**Figure 5:**
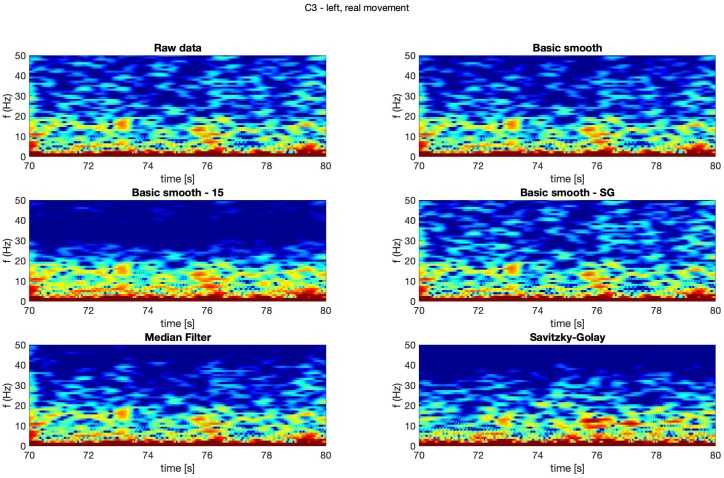
Spectrogram—C3, left hand, real movement.

**Figure 6:**
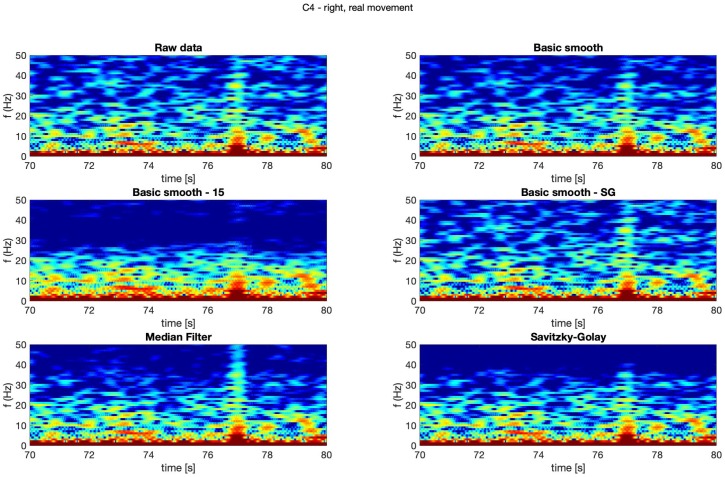
Spectrogram—C4, right hand, real movement.

**Figure 7:**
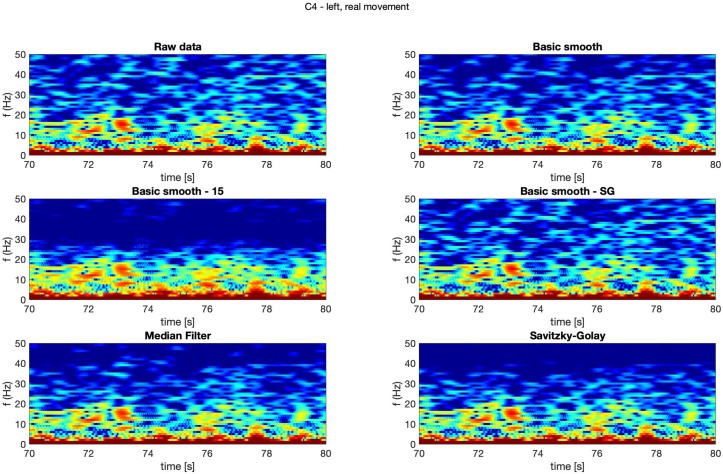
Spectrogram—C4, left hand, real movement.

In Figure 4, Figure 5, Figure 6 and Figure 7 spectrograms (10 seconds intervals) of filtered and raw data for real, both hand movements (channels: C3 and C4) are presented.

The spectrograms presented in Figure 4, Figure 5, Figure 6 and Figure 7 prove that the implementation of the Savitzky–Golay filter and median filter gave the most legible results, with the small advantage of the median filter.

Similar observations where made while analysing of imagery movement data.

**Figure 8:**
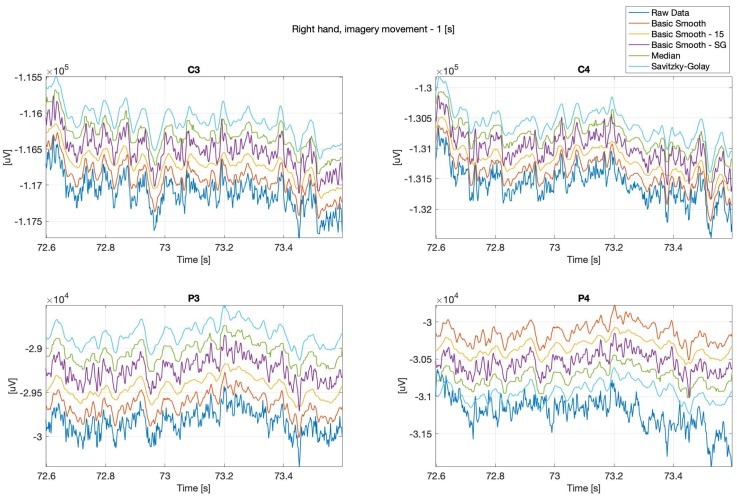
Right hand, imagery movement, C3, C4, P3 and P4 channels.

**Figure 9:**
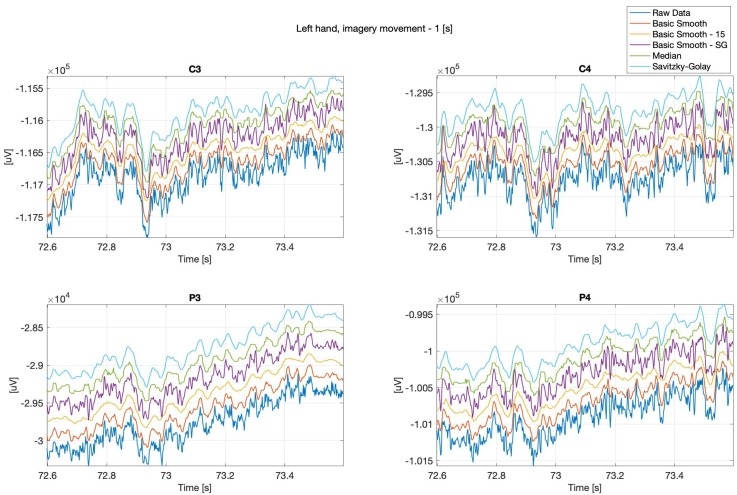
Left hand, imagery movement, C3, C4, P3 and P4 channels.

The data comes from the recording performed during imagery movements, which were illustrated with Figure 8 and Figure 9.

**Figure 10:**
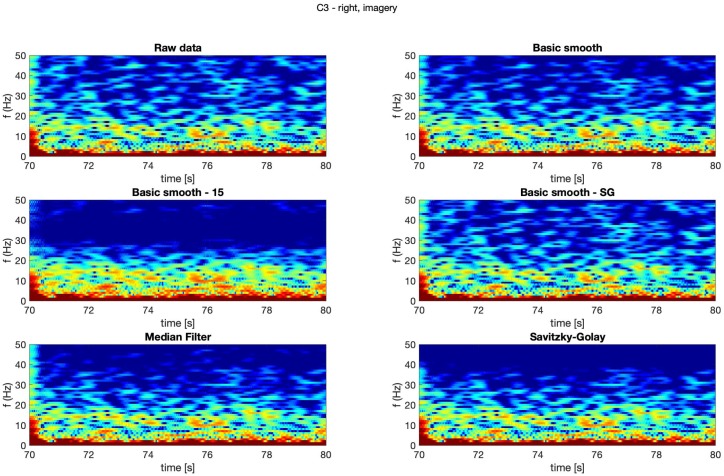
Spectrogram—C3, right hand, imagery movement.

**Figure 11:**
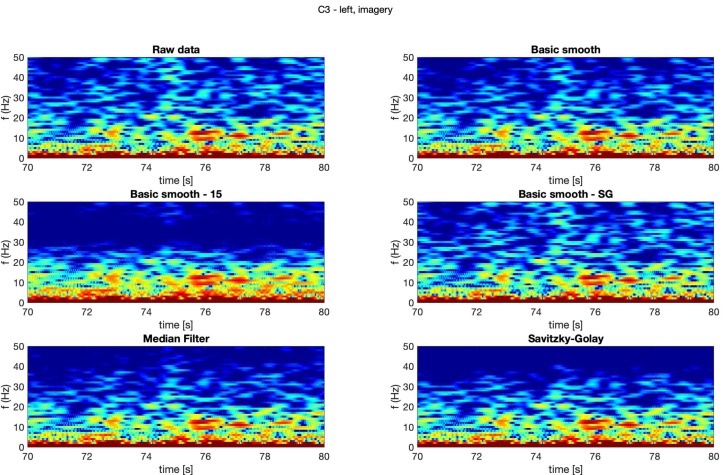
Spectrogram—C3, left hand, imagery movement.

**Figure 12:**
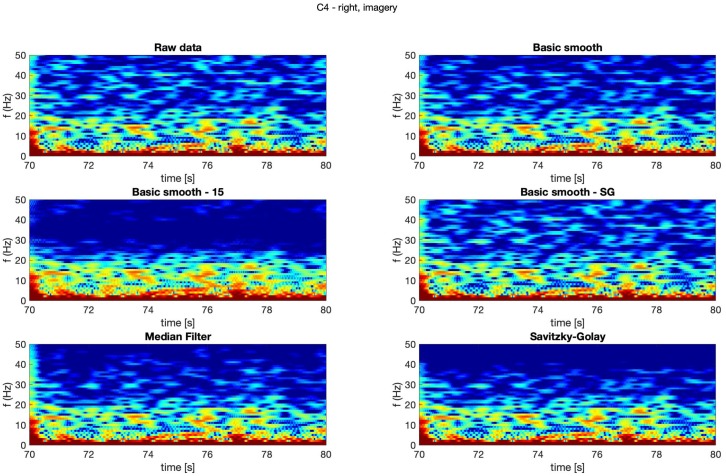
Spectrogram—C4, right hand, imagery movement.

**Figure 13:**
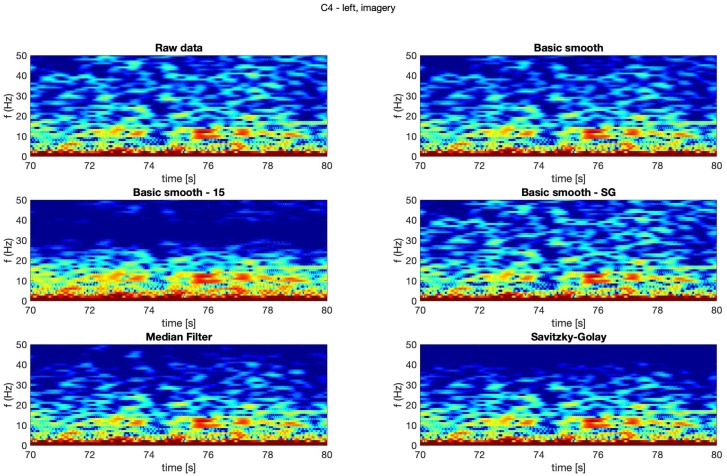
Spectrogram—C4, left hand, imagery movement.

The thorough analysis of the obtained spectrograms (Figure 10, Figure 11, Figure 12 and Figure 13) of the imagery movement data gave similar results to the those received during analysis of the real movements.

**Figure 14:**
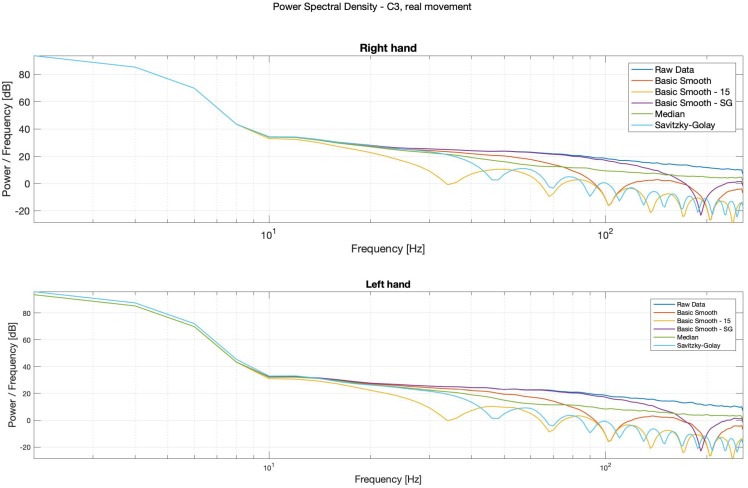
PSD—Bode plots, C3, real movement, right (**top**) and left (**bottom**) hands.

**Figure 15:**
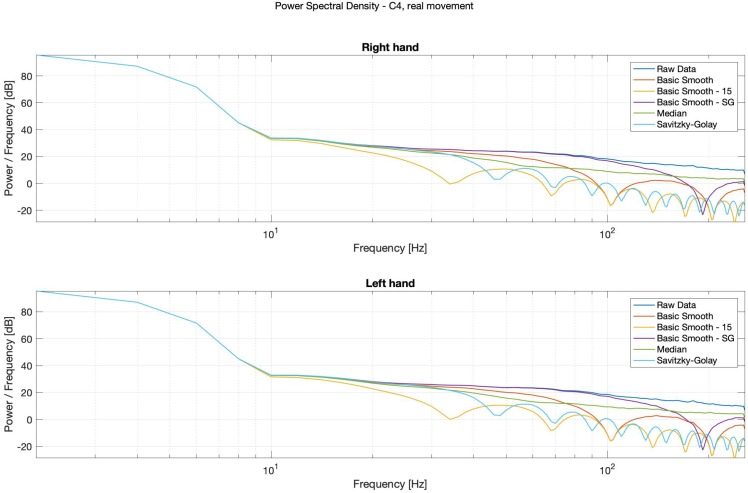
Power Spectral Density (PSD)—Bode plots, C4, real movement, right (**top**) and left (**bottom**) hands.

**Figure 16:**
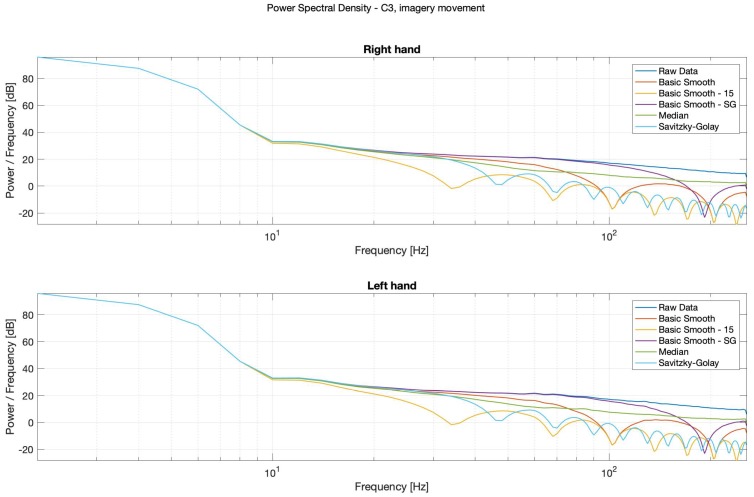
PSD—Bode plots, C3, imagery movement, right (**top**) and left (**bottom**) hands.

**Figure 17:**
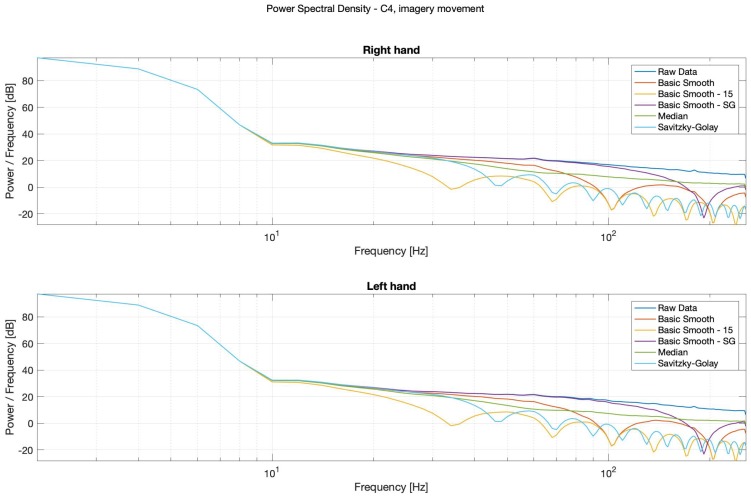
PSD—Bode plots, C4, imagery movement, right (**top**) and left (**bottom**) hands.

It is possible to observe in Power Spectral Density (PSD) diagrams (see: Figure 14, Figure 15, Figure 16 and Figure 17) that the raw data and the median data plots have the most similar shape.

For the potential diagnostics purposes, in order to inter alia observe the alpha waves, without using the spectrograms, the classical, smooth-Savitzky–Golay filter gave the most promising results. It visibly smoothed the data, but did not distort the waves’ shapes.

## 4. Discussion

Appropriate choice of filtering may improve various solutions requiring using the EEG data and may improve advance in the development of brain–computer Interfaces. It is an excellent way for communication for handicapped users [6,18,20,23,48].

It may also help in finding appropriate markers for particular psychiatric disorders. Unfortunately, in current psychiatry the interview is still the main diagnostics tool. It makes it hard for the psychiatrist to choose the appropriate treatment method. The EEG-based diagnosis may support this choice [19,20]. Those methods can help explain the role of cortico-subcortical loops not only in the perspective of physiological control of the majority of motor, emotional and cognitive functions [49] but as well explanation of mental disease pathophiology (among others: addiction, schizophrenia, bipolar disorders) and brain structures sources of mind [50]. Multiple scientific groups are currently working on finding neurophysiological biomarkers of various psychiatric disorders [19].

The study of the EEG data could be performed as an index of training-related brain plasticity in the motor cortex. Due to plastic changes, the EEG could indirectly but objectively reveal changes in cerebral activity related to physical training. This method could be used as a future diagnostic test in the follow-up of patients undergoing rehabilitation. It could also have potential applications in the fields of sports medicine [51].

Abnormal power and functional coupling of resting-state observed in cortical EEG rhythms can also be used to predict and monitor the evolution of Alzheimer’s disease and its relative impact on cognitive domains in pre-clinical, prodromal, and dementia stages of Alzheimer’s disease [52].

### Further Research Plans

The authors of this work are planning to apply non-integer order filters and to compare them with the above-mentioned filters with fractional filters. Non-integer order filtering implementation in the analysis of biomedical data is still an innovative and uncommon idea [3,4], but the theoretical background of fractional systems has a much longer history [8,53]. At first, it was mentioned in a letter to L’Hospital in 1695. The first contributions to the topic were made already in the 18th century by Euler and Lagrange and the first studies on it were performed in the 19th century by Liouville, Riemann and Holmgren [54]. The theoretical basis of non-integer order filters has been very well documented in various publications (such as inter alia: [55]), but its use in bio-medical field is still new and not fully discovered [8,53,56].

The use of non-integer filters in the processing of biomedical signals is becoming more and more popular [3,4,8,53,56,57,58]. This is especially in regards of analysis of EEG, EMG or ECG), as it was mentioned above [13,14,56].

Obviously, fractional filters may appear very useful in many application domains out of which the widely understood smart/autonomous (control) systems seem to be extremely prospective. The main reason is that in such systems the awareness of the surrounding environment, which is essential to implement any kind of autonomous behavior, is usually coming from various kinds of sensors. The data provided by the sensors, even for the most sophisticated ones that usually are equipped by their manufacturers with built-in filtration capability, is usually very noisy and may need to be, not only properly filtered, but also additionally compensated (e.g., gyroscope data/drift widely applied to UAVs) [59].

## 5. Conclusions

The overwhelming significance of the knowledge of basic elements of electroencephalography in its application to the diagnostic workup and the management of patients with suspected or already established generalized epilepsy (GE), however, there is a dearth of data on the pattern and utility of clinical variables that can independently determine EEG abnormalities in GE. The paper [60] underscores the relevance of the different parameters used to decode movement, using EEG in severely paralyzed stroke patients.

Further implementation of proposed filtering methods was shown in the aspect of smoothing inverted pendulum’s movement trajectories. The filtering was implemented for the purpose of some artifacts removal. The authors have already tested various classical smoothing filters on the single-inverted pendulum (a classical problem in control theory) [61,62,63].

However, the filtration may not satisfy the deployment platform requirements and additionally, if needed, it may be difficult to change or tune the filtration parameters to satisfy the desired filtration quality. This is why it is usually possible to access the raw data in order to potentially implement some alternative filtration methods. This space can easily and efficiently be filled out by the fractional filters that provide exceptionally good frequency selectiveness so much needed for the decision-making process (which is an integral part of autonomous behavior). Typically, the technologies that are applied to smart/autonomous systems (e.g., artificial neural networks, fuzzy logic, policy-based computing, or some combinations of all or some of them [64], etc…) rely on the data sensed from the systems processing them in the “as they are” form. It is easy to understand that the better is the sensed data quality, the more accurate are the decisions made by the system.

**Figure 18:**
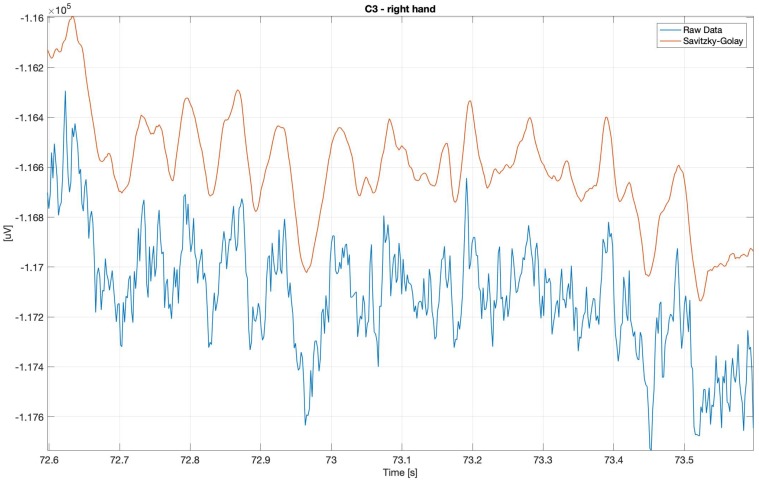
Raw and Savitzky–Golay filtered electroencephalography (EEG) data—clearly visible ‘alpha’, imagery movement.

As was mentioned above, the authors found that the classical, smooth-Savitzky–Golay filter gave the most promising results. It visibly smoothed the data, but did not distort the waves’ shapes. In Figure 18 below, it is possible to observe clear ‘alpha waves’ in 1 second period when the signal was filtered using the Savitzky–Golay Filter. The raw data is very spiky and full of artifacts, although the spectrograms proved the strong presence of the alpha waves.

Using smoothing filters in analysis of the EEG data makes them more legible in aspect of the waves’ shapes. It also does not require using spectrograms.
